# Departing With the Wind: Spring Migration Timing in Brent Geese From Their Most Important Staging and Wintering Site, the Wadden Sea World Heritage Site

**DOI:** 10.1002/ece3.74119

**Published:** 2026-07-31

**Authors:** Nora Theurich, Stefan Garthe, Frédéric Jiguet, Pierrick Bocher, Philipp Schwemmer

**Affiliations:** ^1^ Research and Technology Centre (FTZ), Kiel University Büsum Germany; ^2^ UMR7204 CESCO, Museum National D’Histoire Naturelle, CNRS Sorbonne Université Paris France; ^3^ Littoral Environnement et Sociétés (LIENSs) UMR 7266 La Rochelle University – CNRS La Rochelle France

**Keywords:** Cox proportional hazards, departure timing, East Atlantic Flyway, migratory departure decisions, wind‐mediated movement

## Abstract

The timing of migratory departure represents a critical behavioural decision for long‐distance migrants, influencing arrival condition at breeding grounds and reproductive success. Despite growing recognition of wind as a proximate departure cue, the relative importance of multiple meteorological variables, and whether wind selectivity changes over the departure window, remain poorly quantified at the individual level for most species. We examined meteorological drivers of individual spring migration departure in Dark‐bellied Brent Geese ( *Branta bernicla bernicla* ) from the German Wadden Sea, the species' principal staging area along the East Atlantic Flyway. GPS tracking data from 43 individuals contributing 47 departure events across seven seasons (2015–2024) were combined with hourly meteorological data extracted at each bird's staging location and analysed using Cox proportional hazards models across 18,036 hourly risk intervals. The best‐supported model combined wind assistance at 100 m altitude—consistent with the median sustained flight altitude of 69 m—and atmospheric pressure, with departure probability increasing by 20% per additional m/s of tailwind (HR = 1.20, 95% CI 1.12–1.29) and 10% per additional hPa (HR = 1.10, 95% CI 1.06–1.15). Relative humidity was a competitive secondary predictor, with higher humidity reducing departure probability (HR = 0.959 per %, 95% CI 0.932–0.986). Time‐varying analyses revealed that wind selectivity was strongest early in the season (HR ≈ 1.69) and declined to near unity by early June (HR ≈ 0.98), reflecting increasing migratory urgency. Atmospheric pressure showed no time‐varying effect, acting as a stable synoptic quality filter rather than an urgency‐modulated cue. These findings demonstrate that Brent Geese fine‐tune spring migration onset by integrating local wind conditions and synoptic pressure to exploit transient atmospheric opportunities, highlighting the potential vulnerability of this migration system to projected shifts in North Atlantic wind regimes under ongoing climate change.

## Graphical abstract

The timing of migratory departure reflects a balance between endogenous schedules and transient environmental opportunities. By linking GPS‐derived departure events with hourly atmospheric data, we demonstrate that wind assistance strongly increases departure probability in Brent Geese, while this selectivity weakens over time as migratory urgency increases. Our findings highlight general decision rules by which animals adjust behaviour under changing environmental conditions.

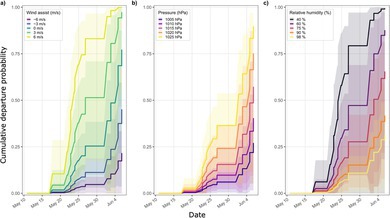

## 1 Introduction

Migration timing in long‐distance birds reflects a balance among evolutionary pressures. Early arrival at the breeding grounds can secure high‐quality territories and synchronise reproduction with peak food availability but carries the risk of encountering adverse conditions en route or upon arrival (Clausen and Clausen 2013; Lameris et al. 2018). Conversely, delayed departure may result in suboptimal nesting sites—as prime territories are claimed by earlier arrivals (Kokko 1999)—missed peaks of resource availability, and a shorter period for chick development, constraints that are especially critical for Arctic breeders in areas with short summers (Bety et al. 2004; Doiron et al. 2015; Lameris et al. 2017). Late arrival has accordingly been linked to reduced reproductive success in geese, particularly those breeding in the high Arctic (Lameris et al. 2017). Arriving too early, however, can also be costly. When conditions remain unsuitable, early migrants may experience delays before initiating breeding. This is particularly relevant in Arctic‐breeding geese, which are at least partly capital breeders and thus depend on body reserves accumulated before arrival to support reproduction. For example, Barnacle Geese ( *Branta leucopsis* , Bechstein 1803) that advance northwards in years of early snowmelt often need to rebuild body reserves before egg laying (Lameris et al. 2018). The timing of migration departure is thus shaped by strong selective pressures, and migratory birds are expected to fine‐tune the initiation of their journeys to maximise fitness. Because body reserves take time to accumulate, individual body mass at the end of the staging period may itself signal readiness to depart, with heavier individuals—having secured sufficient reserves—potentially able to leave earlier, while lighter individuals may delay departure to continue provisioning. Sex‐specific differences in departure timing, by contrast, are less expected in this species, as Brent Geese typically migrate in stable pair bonds and family groups, with partners departing together rather than on independent schedules.

**FIGURE 1:**
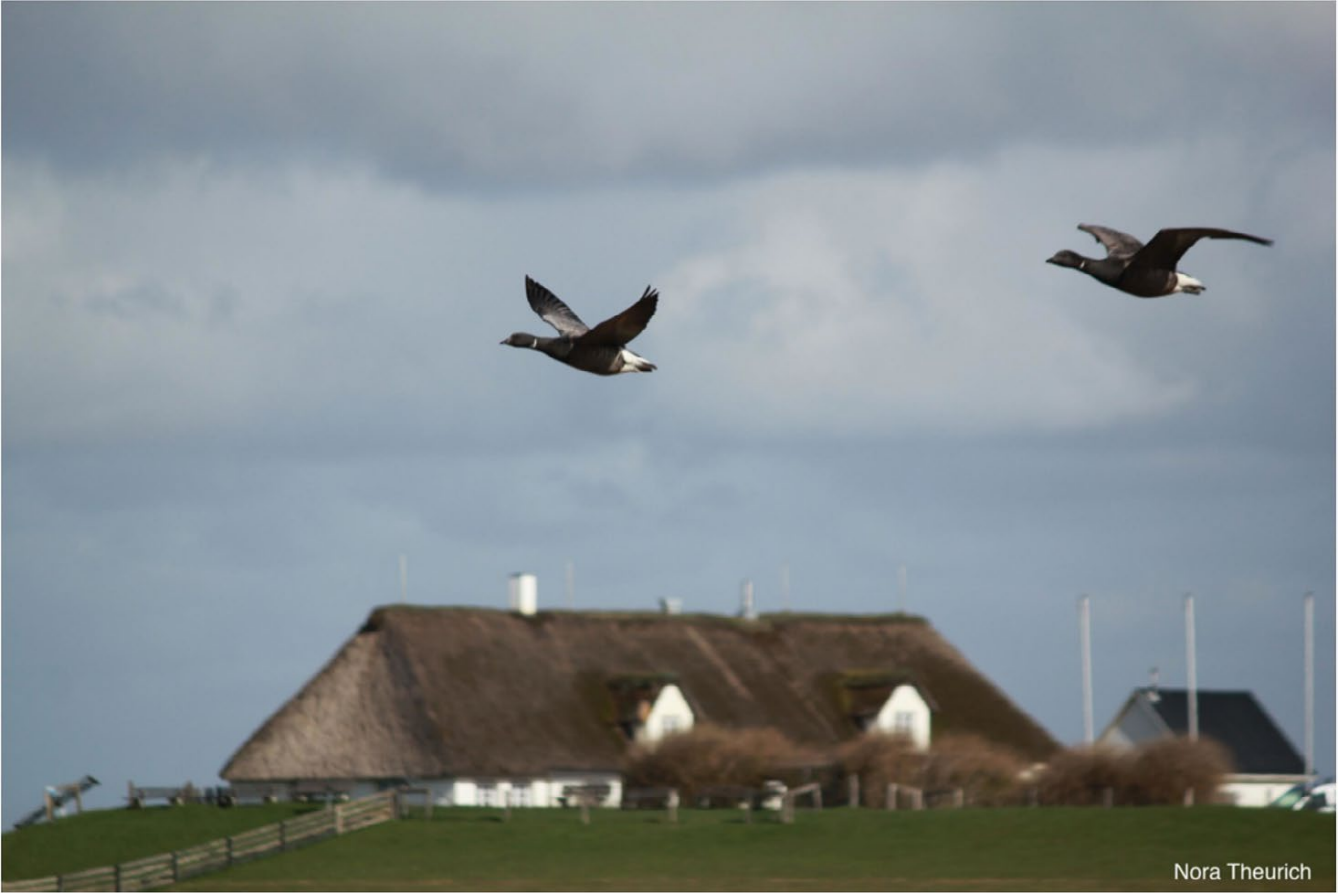
The study species, Dark‐bellied Brent Goose ( *Branta bernicla bernicla* ), photographed at Hamburger Hallig in the German Wadden Sea.

Although the broad seasonal schedule of migration is largely determined by endogenous rhythms and photoperiod (Berthold 2001; Gwinner 2003), proximate environmental cues govern the fine‐scale, daily decisions of when to commence migration (Newton and Brockie 2008). Among these cues, weather, particularly wind, has long been recognised as a key external trigger of migratory departure (Bruderer 1992; Richardson 1990). Tailwinds increase the ground speed and reduce energetic costs during flight, whereas headwinds slow progress and increase energetic expenditure (Liechti 2006; Pennycuick 1996). Although headwinds can support climbing flight in some soaring species (Leshem and Yom‐Tov 1996), which is potentially advantageous for crossing barriers, Dark‐bellied Brent Geese ( *Branta bernicla bernicla* , Linnaeus 1758; hereafter Brent Geese, Figure 1) typically travel at low altitudes in flapping flight and are therefore expected to benefit primarily from tailwind support. Consequently, many species delay departure until favourable winds arise, and both theoretical and empirical studies support the concept of wind‐assisted departure windows (Åkesson and Hedenström 2000; Liechti and Bruderer 1998; Loring et al. 2020). Wind selectivity, however, varies throughout the migration window: migrants tend to rely most on favourable winds early in the season, when short delays can be accommodated, and become less selective as the internal timing constraints intensify (Åkesson and Hedenström 2000). Whether this predicted decline in wind selectivity occurs in Brent Geese, and whether it reflects a hardened internal motivation to depart or simply a reduction in available high‐quality departure windows later in the season, has not previously been tested with individual‐level data. Climatological analyses of shorebird migration have shown that migrants departing from the Wadden Sea for Siberia frequently encounter strong tailwinds (Piersma and Van de Sant 1992), suggesting that natural selection may have aligned migration schedules with recurring atmospheric patterns (Hedenström 2008). Birds also respond to additional weather cues, such as rising barometric pressure and the absence of precipitation, which typically accompany clear skies and low‐risk flying conditions (Dokter and Ebbinge 2013; Cooper et al. 2023). Conversely, rainfall can impede flight performance, reduce visibility and challenge thermoregulation (Richardson 1990). Because many of these meteorological variables are interrelated, isolating their independent effects under field conditions is challenging, a limitation we account for through collinearity screening and multi‐model comparison in the present study. Moreover, prior studies have often characterised weather conditions at regional or population level rather than at the precise location of each individual bird, potentially obscuring individual variation in cue perception.

Brent Geese are long‐distance migratory waterfowl that breed in the high Arctic of Siberia, primarily on the Yamal and Taymyr Peninsulas and on islands along the western Siberian coast (Ebbinge and Spaans 1995; Green et al. 2002). The population studied here winters along the East Atlantic Flyway, with major non‐breeding sites along the German North Sea coast, the English Channel and the French Atlantic coast. The dark‐bellied subspecies numbers approximately 200,000–280,000 individuals (BirdLife International 2024) and is listed under Annex I of the EU (European Union) Birds Directive and protected under the African‐Eurasian Migratory Waterbirds Agreement (AEWA). In spring, nearly the entire population gathers in the Wadden Sea World Heritage Site (Kleefstra et al. 2019)—the sole major pre‐breeding staging area along the flyway—where birds accumulate large fat reserves before initiating their northward migration (Ebbinge and Spaans 1995; Green et al. 2002). Unlike some other waterfowl that migrate earlier in the season, Brent Geese delay their departure and then migrate in a tightly synchronised fashion, leaving the Wadden Sea within a short period in late May and early June, likely timed to coincide with Arctic snowmelt and the brief breeding season (Green et al. 2002). Earlier satellite studies from the Wadden Sea suggested that departures and early migration phases occurred under tailwind conditions, but those datasets relied on regional weather station data and a small number of tracked individuals and did not formally analyse wind as an individual‐level proximate cue (Green et al. 2002). This pattern mirrors behaviours in other Arctic‐breeding geese, such as Greater White‐fronted Geese ( *Anser albifrons* , Scopoli 1769) and Barnacle Geese, which use combinations of photoperiod, seasonal patterns of food availability and weather cues to schedule their migrations (Kölzsch et al. 2015; Shariati‐Najafabadi et al. 2016; van Wijk et al. 2012). Because Brent Geese cross the Baltic Sea—where eight bordering nations have committed to expanding offshore wind capacity from approximately 3 GW to nearly 20 GW by 2030 (WindEurope 2022), understanding how wind influences their migration decisions is an issue of growing conservation relevance. Identifying the environmental cues that trigger departure and shape flight timing can inform risk assessments and guide spatial planning to minimise collision risks—not only with offshore wind turbines (Schwemmer et al. 2023), but also with aircraft in busy airspace corridors along the flyway (van Gasteren et al. 2019). Field observations and GPS tracking have repeatedly shown that mass departures of Brent Geese coincide with shifts in wind direction, suggesting that wind assistance acts as a decisive external cue. In some years, tens of thousands of birds have been observed departing within 48 h of a switch to favourable winds, and individual‐level tracking confirmed that Brent Geese rarely initiated migration under headwind conditions (Green et al. 2002). However, favourable winds represent only one of several environmental factors that may shape departure timing, and their relative importance compared with other weather conditions—including atmospheric pressure, humidity and temperature dynamics—has not yet been formally quantified for this species.

Despite this body of work, three specific questions remain unresolved for Brent Geese departing the Wadden Sea. First, the relative contribution of multiple simultaneous meteorological variables—including wind assistance, atmospheric pressure, humidity and temperature dynamics—to individual departure decisions has not been formally quantified; previous studies identified wind as a general correlate but did not test competing cues at the individual level. Second, whether wind selectivity changes over the course of the spring departure window, consistent with time‐minimisation theory (Åkesson and Hedenström 2000), has not been tested explicitly with individual‐level data for this species. Third, prior analyses used regional or population‐level weather data rather than the conditions experienced at each individual's precise staging location, potentially obscuring individual variation in cue perception. The present study addresses all three gaps.

Using high‐resolution GPS data from 43 individuals contributing 47 departure events across seven spring seasons within 2015 to 2024, we identified the precise timing of departure for each individual and linked those events to contemporaneous weather conditions extracted at each bird's GPS‐defined staging location. We sought to disentangle the relative importance of local atmospheric drivers—wind assistance and humidity conditions at the staging site—and large‐scale synoptic conditions—atmospheric pressure—on individual departure decisions. Local weather may act as the immediate trigger for take‐off, whereas regional pressure systems reflect the broader suitability of the synoptic environment for migration. Specifically, we asked which meteorological variables were most strongly associated with individual departure decisions during the spring migration window, and whether wind selectivity changed over the course of that window. Because the broader seasonal timing of migration is likely constrained by endogenous rhythms and photoperiod, we focused on the environmental cues associated with the exact timing of take‐off within that window. We hypothesised that departure timing would be primarily governed by meteorological cues, particularly wind assistance, with stronger reliance on favourable winds early in the departure window before internal motivation to depart intensified. We tested this by modelling departure probability as a function of environmental variables using Cox proportional hazards models, incorporating wind assistance as the hypothesised primary predictor alongside additional meteorological variables, and applying time‐varying coefficient extensions to test explicitly whether wind selectivity declined over the season.

## 2 Methods

### 2.1 Study Area and Tagging of Brent Geese

**FIGURE 2:**
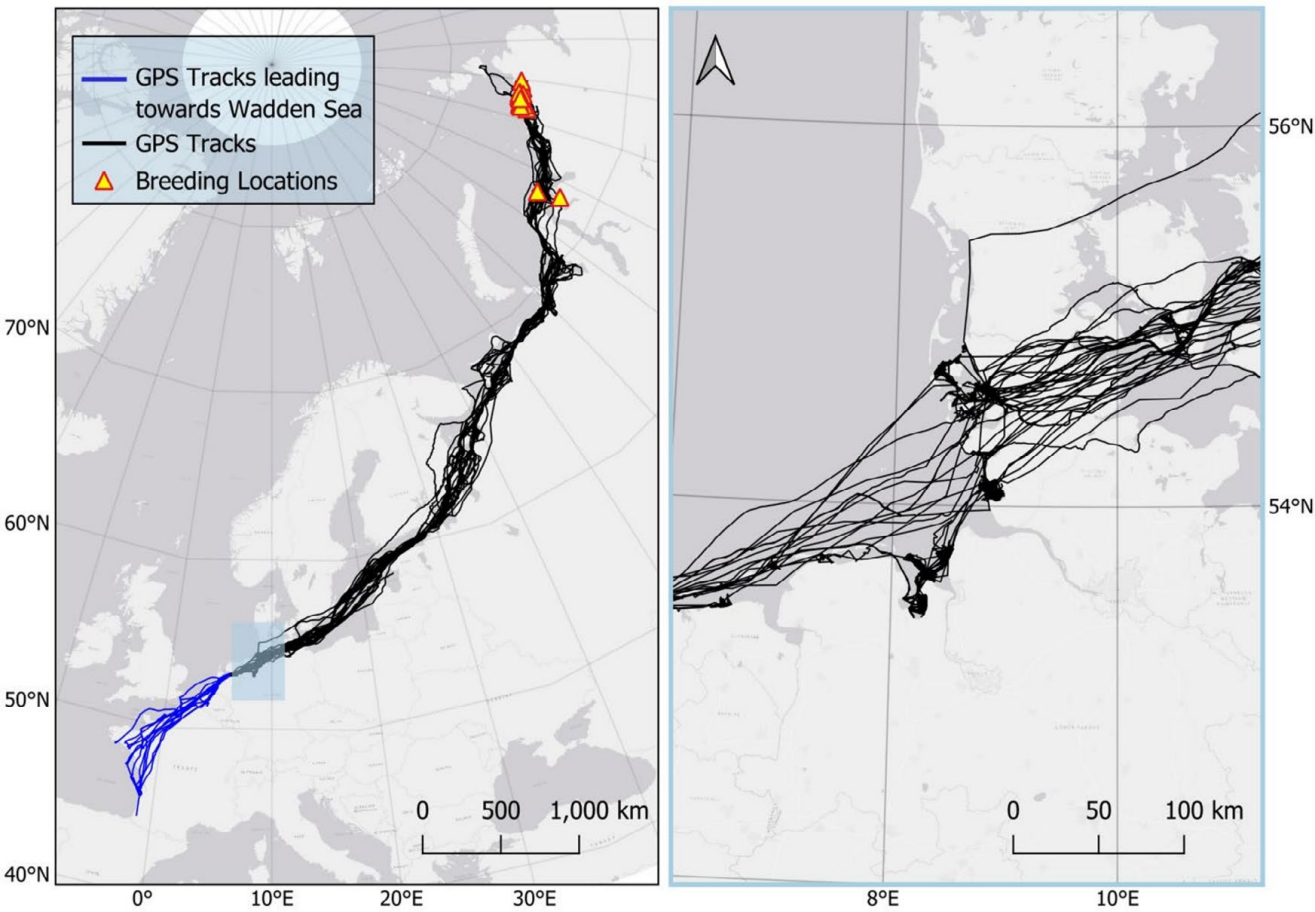
Spring migration routes and departure locations of Brent Geese ( *Branta bernicla bernicla* ) tracked between 2015 and 2024. Left panel: Migration trajectories of 47 individual‐year tracks from 43 unique individuals originating from wintering grounds in Western Europe and terminating at breeding areas in north‐western Russia. Tracks leading to the Wadden Sea staging shown in blue; outbound spring migration from the Wadden Sea shown in black; yellow triangles indicate Arctic breeding locations. Right panel: Enlarged view of spring departure sites along the German Wadden Sea.

We obtained high‐resolution GPS tracking data from 61 adult Brent Geese between 2015 and 2024. All individuals were equipped with solar‐powered GPS transmitters (Ornitela OT‐20, OT‐25, or OT‐30; 20–30 g, Ornitela, UAB, Lithuania), which were attached using a Teflon‐ribbon body harness in a backpack configuration, following established procedures (Thaxter et al. 2014). The tag mass represented < 3% of individual body mass (mean = 1442 g; range = 971–1812 g). Birds were captured during discrete trapping sessions using cannon nets, mist nets, or foot slings. Of the 61 individuals, 40 were tagged along the eastern Wadden Sea coastline in Schleswig‐Holstein and northeastern Lower Saxony (Germany), whereas 21 adults originated from a French project (https://migratlane‐telemetrie.fr). French birds were tagged at different sites along the Atlantic coast. Although the capture protocols differed slightly between the projects, all birds were processed according to comparable standards: standard biometrics were measured to the nearest millimetre and birds were weighed to the nearest gram and fitted with metal rings. Feather samples were collected from the 40 birds tagged by Kiel University Research and Technology Centre (FTZ) for genetic sex determination (Tauros Diagnostics, Bielefeld, Germany). Sex for French‐tagged individuals was determined in the field via cloacal examination. GPS data from all tags were transmitted via the Global System for Mobile Communication (GSM) network and archived in the Movebank repository (www.movebank.org). For the present analysis, we only retained data for individuals that initiated spring migration via the Wadden Sea (see right panel map in Figure 2), including French‐tagged birds that typically spent several weeks in the area before continuing towards Arctic breeding grounds. Eighteen individuals did not contribute to the final departure dataset because no reliable spring departure event could be assigned, for example due to transmitter loss, non‐migratory behaviour, incomplete tracks, long temporal gaps, or ambiguous movement patterns. The final dataset comprised 43 individuals and 47 individual‐year departure records, because four individuals contributed data in 2 years. Of these 43 individuals, 15 were male, 23 were female and sex was undetermined for five individuals.

### 2.2 Processing of GPS Data and Departure Classification

All tracks were inspected visually in QGIS (version 3.44.0, QGIS Development Team 2025) using Esri satellite basemaps and shapefiles outlining known breeding regions on the Taimyr Peninsula, West Siberian islands and the Yamal Peninsula (Ebbinge and Spaans 1995; Green et al. 2002). For display purposes in the migration map, breeding locations were identified algorithmically using the package *dbscan* (Hahsler et al. 2019), selecting the northernmost cluster with the longest stay duration as each individual's putative breeding site. Tracks from individuals that failed to initiate spring migration, for example, due to transmitter loss or non‐migration behaviour, were excluded. Only tracks with a mean GPS fix interval < 100 min (mean = 17 min) and without temporal gaps > 24 h between 10th May and the first 24 h post‐departure were retained. Subsequent analyses were conducted in R (version 4.6.0, R Core Team 2025). For birds tracked in multiple years, each year was treated as a separate individual‐year record, and this identifier was included as the clustering variable in all analyses. Spatial and temporal displacement between GPS fixes were calculated using the Haversine method implemented in the *geosphere* package (Hijmans 2022). From 84 candidate tracks, 47 were confirmed as true spring departures. All individuals were tagged at least 10 days prior to departure, with most marked > 1 month in advance and some up to 5–6 months earlier. This substantial delay between handling and departure ensured that the departures analysed here were not artefacts of recent capture activity.

To separate flight from non‐flight behaviour, step speeds were calculated from consecutive GPS fixes, and the speed distribution was inspected for bimodality. Low‐speed and high‐speed modes were identified, and the classification threshold was set at the local minimum between these peaks. Using this threshold (7.38 m/s), only flight‐classified points were retained for subsequent flight‐height analyses. Altitude data were provided by the Ornitela GPS transmitters and filtered to remove biologically implausible values. Because GPS‐altitude measurements near sea level can produce negative values during low flight, endpoint heights between −25 and 0 m were retained and recoded to 0 m; segments with either endpoint height below −25 m were excluded. Segment height was calculated as the mean of the two adjusted endpoint heights and summarised in height classes rather than treated as a precise continuous measurement. Class proportions were calculated per individual‐year and averaged across individual‐years to avoid over‐weighting individuals with many fixes.

Departure events were defined as the initiation of the first sustained migratory movement that met three simultaneous criteria: (i) a minimum displacement of 30 km in a consistent direction; (ii) a step speed exceeding 7.38 m/s, a threshold below which movements were consistently attributable to local foraging or within‐stopover activity rather than directed flight; and (iii) crossing the 9°E meridian within 24 h after the detected departure—which served as a standardised spatial benchmark, as it was the first common longitude crossed by all individuals exclusively during post‐departure migration and never during preceding staging movements within the Wadden Sea. The precise departure timestamp was assigned to the last recorded non‐flying GPS fix within the Wadden Sea prior to the first qualifying migratory step crossing 9°E, representing the final confirmed staging position before the onset of spring migration. To characterise diel departure structure and determine whether time‐of‐day stratification was warranted in subsequent models, departure times were visualised across 24‐h intervals in UTC. Mean sunrise and sunset times for the departure period were calculated using the *suncalc* package (Thieurmel and Elmarhraoui 2017), to identify whether departure peaks coincided with phases of the light cycle.

### 2.3 Meteorological Data

Meteorological predictors were obtained from the Open‐Meteo historical weather API (Zippenfenig 2023), which provides hourly atmospheric fields derived from ERA5 reanalysis (European Centre for Medium‐Range Weather Forecasts) at 0.25° spatial resolution (approximately 17 × 28 km at the study latitude). Values were extracted at the corresponding grid cell for each individual's GPS‐recorded departure location, providing bird‐specific hourly meteorological conditions rather than broader regional averages. Departure coordinates ranged from 54.1–54.6°N, 8.8–8.97°E along the eastern Wadden Sea coast. The following variables were considered: wind speed and direction at 10 m and 100 m altitude, 2 m air temperature, dew‐point temperature, sea‐level pressure, total cloud cover and precipitation. Wind assistance was computed at both 10 and 100 m altitude and compared in formal model selection.

Wind assistance was calculated as the wind component in the mean migratory direction (47°):

$$\text{Wind assistance}=u\times \sin\;\left(\theta \right)+v\times \cos\;\left(\theta \right)$$

where *u* and *v* are the zonal and meridional wind components (in m/s), and *θ* is the migratory bearing in radians. To capture short‐term atmospheric dynamics, trend variables were derived as slopes from ordinary least‐squares regressions (OLS) over moving windows applied to each individual's weather series. Window lengths were matched to the characteristic timescale of each variable: 24 h for atmospheric pressure, to capture synoptic‐scale change while avoiding confounding with the diurnal pressure cycle, and 12 h for wind assistance and dew‐point temperature, reflecting the shorter timescales over which these variables respond to frontal passage. Only slopes significant at *p* ≤ 0.05 were retained to ensure that trends represented meaningful directional changes rather than random variation. Wind conditions at departure hours were summarised using wind‐rose plots based on the openair package (Carslaw and Ropkins 2012) and compared against the background distribution of all available hours within the departure window to confirm selective departure under favourable conditions.

### 2.4 Statistical Analysis

Prior to any weather‐related departure modelling, we assessed the effects of sex, body mass and wingspan on departure date using ANOVA and linear regressions respectively. Weather dependence of departure timing was analysed using Cox proportional hazards models (Therneau and Grambsch 2000) in a counting–process framework, beginning on 10 May each year. This start date preceded the earliest observed departure while remaining within the typical spring migration window. Each individual‐year contributed hourly risk intervals from 10 May 00:00 UTC up to its observed departure time, with each interval assigned the meteorological conditions prevailing at the start of that hour. In our context, a hazard ratio (HR) > 1 indicated an increased instantaneous departure probability per one‐unit increase in the predictor variable, with all other predictors held constant and relative to the non‐parametric baseline hazard at that hour. The non‐parametric baseline hazard in the stratified Cox model absorbs any systematic increase in departure probability over the season, including time pressure driven by approaching breeding deadlines; weather effects are therefore estimated conditional on this background temporal trend, representing the additional influence of meteorological conditions above and beyond seasonal urgency. Sex and body mass were not included as covariates in the Cox models because they predict the day of departure across the season rather than the hour‐by‐hour departure decision within an individual's risk period; both effects are absorbed by the non‐parametric baseline hazard.

Before model fitting, all candidate variables were screened for missingness, near‐zero variance and multicollinearity. Variance inflation factors (VIF) were computed for all candidate predictors using auxiliary regressions, and variables with variance inflation factors > 5 or pairwise Spearman |*ρ*| > 0.7 were excluded. Temperature (2 m) was removed due to extreme collinearity with relative humidity and dewpoint temperature (VIF = 108.89). All remaining variables, including dewpoint temperature, had VIF < 1.3 and were retained as candidates. Precipitation showed little variation during the study period (mean = 0.07 ± 0.06 mm SE, maximum = 2.8 mm; 89% of departure hours recorded zero precipitation) and was not included as a predictor. The final candidate set comprised wind assistance at 100 m, relative humidity, dewpoint temperature, atmospheric pressure, cloud cover fraction and their associated rolling trend variables (Table 2). Cox models were fitted using the *survival* package (Therneau 2024) with Efron ties and cluster‐robust standard errors (cluster = individual_year) to account for temporal autocorrelation within individuals. To incorporate diel variation, the baseline hazard was stratified into four 6‐h intervals: night (00–06 UTC), morning (06–12 UTC), day (12–18 UTC) and evening (18–24 UTC). Wind assistance was confirmed to be only weakly correlated with hour of day across the full risk set (Spearman *ρ* = −0.050), indicating that the stratification approach was sufficient to separate circadian and meteorological signals. Twenty candidate models were fitted, spanning single‐predictor to three‐predictor combinations of wind assistance with each remaining candidate variable. Model selection was guided by Akaike's Information Criterion corrected for small sample size (AIC_c_; Burnham and Anderson 2004); models within ΔAIC_c_ < 2 of the best models were considered equivalent, and where equivalent models differed in parameter count the more parsimonious model was preferred.

Proportional hazards (PH) assumptions were assessed using (i) global and term‐specific Schoenfeld‐residual tests (*cox.zph*, *survival*) and (ii) likelihood‐ratio tests (LRT) comparing each static model with an equivalent time‐varying‐coefficient (TVC) model defined as *tt*(*x*) *= x* × *log*(1 *+ t*), where t denotes analysis time and $\mathrm{tt}\left(x\right)$ is the time‐varying transformation of a covariate *x*. A variable was considered to violate proportional hazards when its term‐specific test was significant (*p* < 0.05) and the TVC model improved fit (ΔAIC_c_ < 0). Variables fulfilling these criteria were visualised via *ggplot2* (Wickham 2016) as time‐dependent hazard ratios HR(*t*) with 95% confidence intervals (CI), illustrating temporal changes in effect strength. Predictors meeting the proportional hazards assumption were retained as static terms and visualised through predicted survival curves *S*(*t*) and corresponding departure probabilities (1 − *S*(*t*)) for representative covariate values. Variables showing minor proportional hazards deviations without meaningful improvement in fit, or with effect sizes too small to influence interpretation, were considered biologically negligible and excluded from the final model set (see Table 2 for the final set of retained variables). As a model‐independent complement to the TVC analysis, individual‐year departure events were divided into terciles by departure time (early, mid, late; *n* = 16, 15 and 16 respectively) based on hours elapsed since 10 May. This comparison tests the same seasonal pattern identified by the TVC models in the simplest possible form: if wind selectivity declines as migratory urgency increases, birds departing later in the season should on average do so under less favourable wind conditions than early‐season birds, whereas variables without a time‐varying effect should show no such trend across terciles. Normality within each tercile was assessed using Shapiro–Wilk tests; because wind assistance and atmospheric pressure departed from normality in the mid‐season group, and relative humidity departed from normality in the late‐season group (all *p* < 0.05), non‐parametric Kruskal–Wallis tests were used to compare wind assistance, atmospheric pressure and relative humidity at the hour of departure across the three timing groups.

## 3 Results

### 3.1 Migration Phenology and Departure Timing

The 43 tracked Brent Geese contributed 47 departure events overall, departed the Wadden Sea within a relatively narrow seasonal window, and initiated their long‐distance migration towards their breeding grounds in northwestern Russia (Figure 2). The first departure was recorded on 14 May (day of year 134) and the latest on 6 June (day of year 157), with a mean departure date of 26 May (±5.2 days; day of year 146; Table 1).

**TABLE 1:** Overview of tracked individuals, years and confirmed spring departures of Brent Geese.

Metric	Value	Description
Initial tagged individuals	61	Adult Brent Geese fitted with GPS–GSM transmitters (2015–2024)
Confirmed departure events	47	From 43 unique individuals; 4 individuals contributed 2 years
Mean departure date	26 May (day of year 146)	Range: 14 May – 6 June
Mean GPS fix interval	17 min	Max. gap allowed: 24 h
Mean 24 h flight distance	683 km	Range: 139–2081 km

Despite minor interannual variation across seven seasons (2015, 2016, 2018, 2019, 2022, 2023, 2024), departure timing was relatively synchronised, with nearly all individuals, including those tagged in France, leaving the Wadden Sea staging area within a 3‐week period. French‐tagged geese typically arrived in the Wadden Sea between late March and early May and remained at the staging site before initiating migration. Only one French‐tagged individual bypassed the Wadden Sea, flying directly to the Baltic Sea before initiating migration from there, but was still within the same temporal window as birds departing from the Wadden Sea. There was no significant difference in departure date found between sexes (ANOVA, *F* (1, 40) = 0.001, *p* = 0.971, 5 birds with unknown sex excluded). Body mass was negatively associated with departure date (linear model, *F*(1, 45) = 8.22, *p* = 0.006) with heavier individuals tending to depart earlier, whereas wing length showed no significant association (linear model, *F*(1, 43) = 0.63, *p* = 0.432).

**FIGURE 3:**
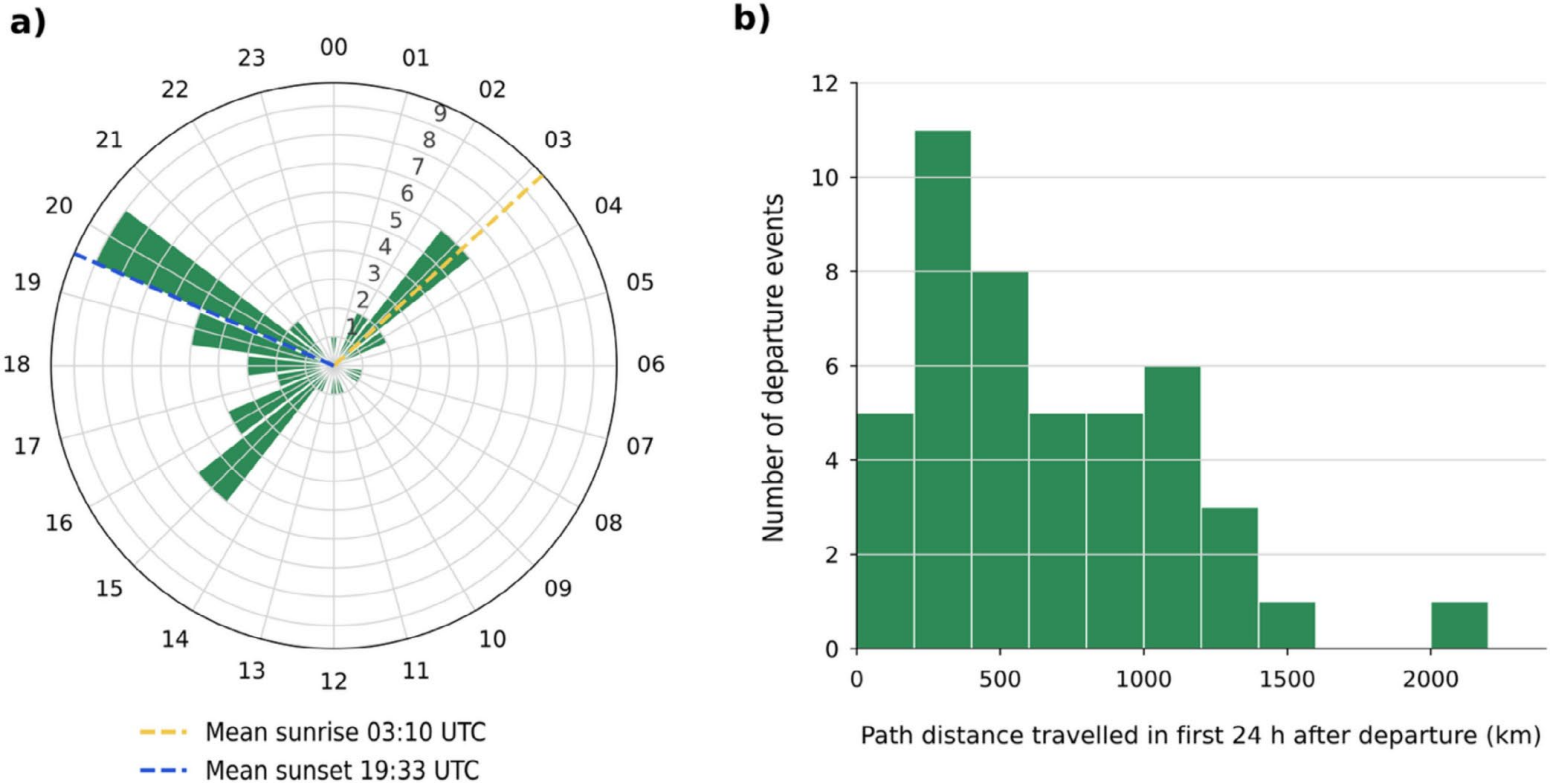
Timing and initial flight distances of spring departures in Brent Geese (*n* = 47). (a) Hour of departure in UTC, with the yellow line indicating mean sunrise time and the blue line mean sunset time for the departure window (14 May–6 June). (b) Distribution of path distances covered within 24 h after departure.

Departure times were not distributed uniformly throughout the day (Figure 3a), with most individuals initiating migration in the late afternoon and evening, typically between 15:00 and 21:00 UTC, with a pronounced peak shortly before sunset around 19:30 UTC. A smaller secondary peak occurred near sunrise, whereas no departures were recorded between 08:00 and 14:00 UTC across the entire dataset, confirming a strong diel structure to departure initiation that was controlled for through time‐of‐day stratification in all subsequent models. Distances travelled within the first 24 h after departure varied markedly among individuals (mean = 683 km ± 446 SD; range 139–2081 km; Figure 3b), with most geese covering 200–300 km during the first day while others initiated rapid long‐distance flights exceeding 1000 km, including one individual that crossed the entire Baltic Sea within 24 h.

### 3.2 Wind Environment

**FIGURE 4:**
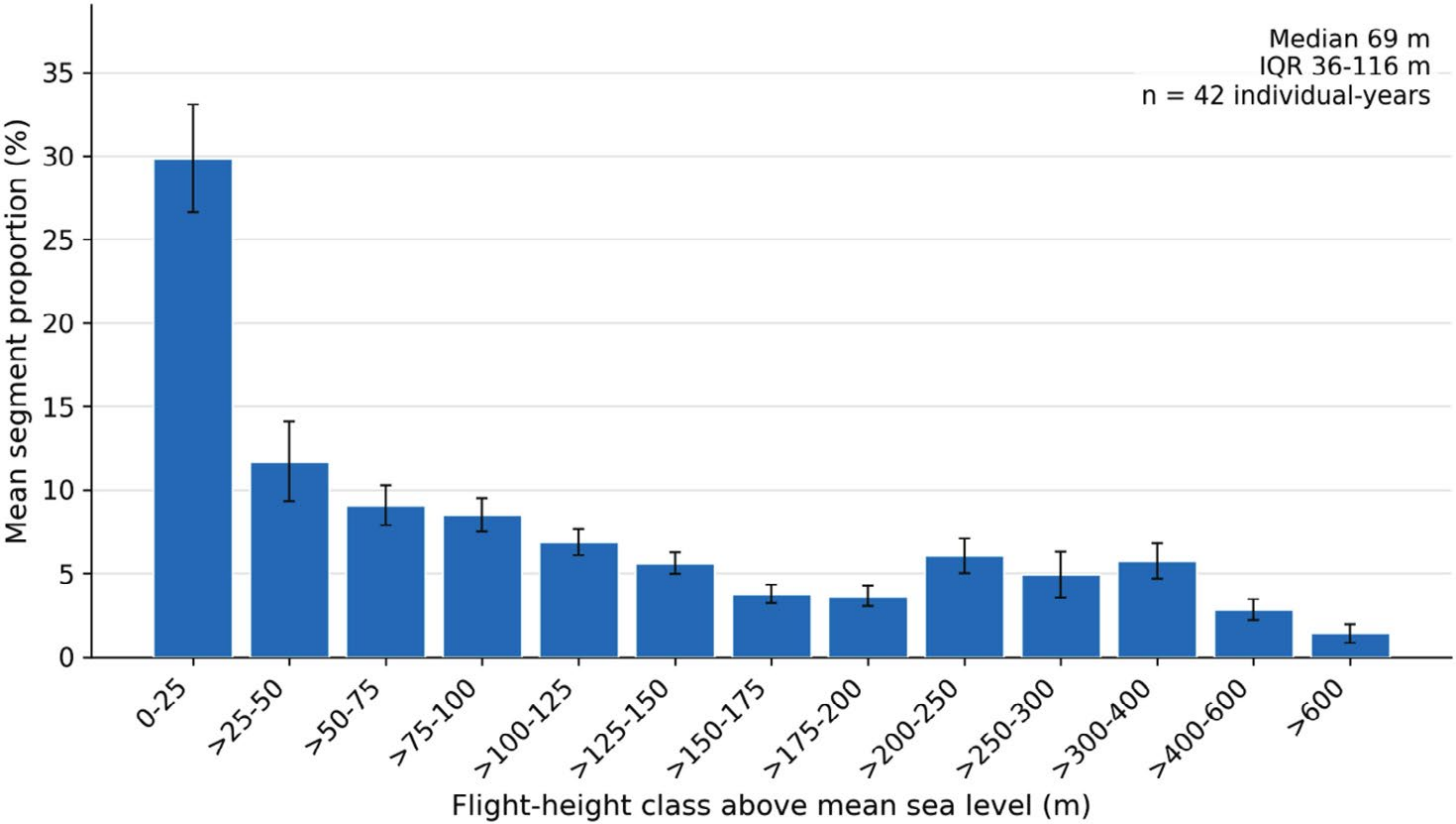
Flight‐height distribution during sustained flight within 24 h of departure. Bars show the mean proportion of flight segments per individual‐year ± SE (*n* = 42 individual‐years with sufficient height data).

Wind assistance was extracted at 100 m altitude, consistent with the median sustained flight altitude of 69 m (IQR 36–116 m) recorded during spring migration (Figure 4). Wind assistance at 10 and 100 m was near‐perfectly correlated across the full staging period (Spearman *ρ* = 0.990, *n* = 31,584 h, *p* < 0.001) and at actual departure hours (*ρ* = 0.970, *n* = 47), confirming that the choice of extraction height had a negligible effect on inference. Sensitivity analyses using 10 m wind assistance produced consistent results (HR = 1.35, 95% CI 1.21–1.50) and had lower AIC_c_ (ΔAIC_c_ = −2.6 relative to the 100 m model), exceeding our pre‐defined equivalence threshold (ΔAIC_c_ < 2). We nonetheless retained 100 m as the primary predictor on biological grounds, as it more closely matches the median sustained flight altitude recorded during the initial spring migration (Figure 4), while both extraction heights yielded near‐identical hazard ratios and identical qualitative conclusions.

**FIGURE 5:**
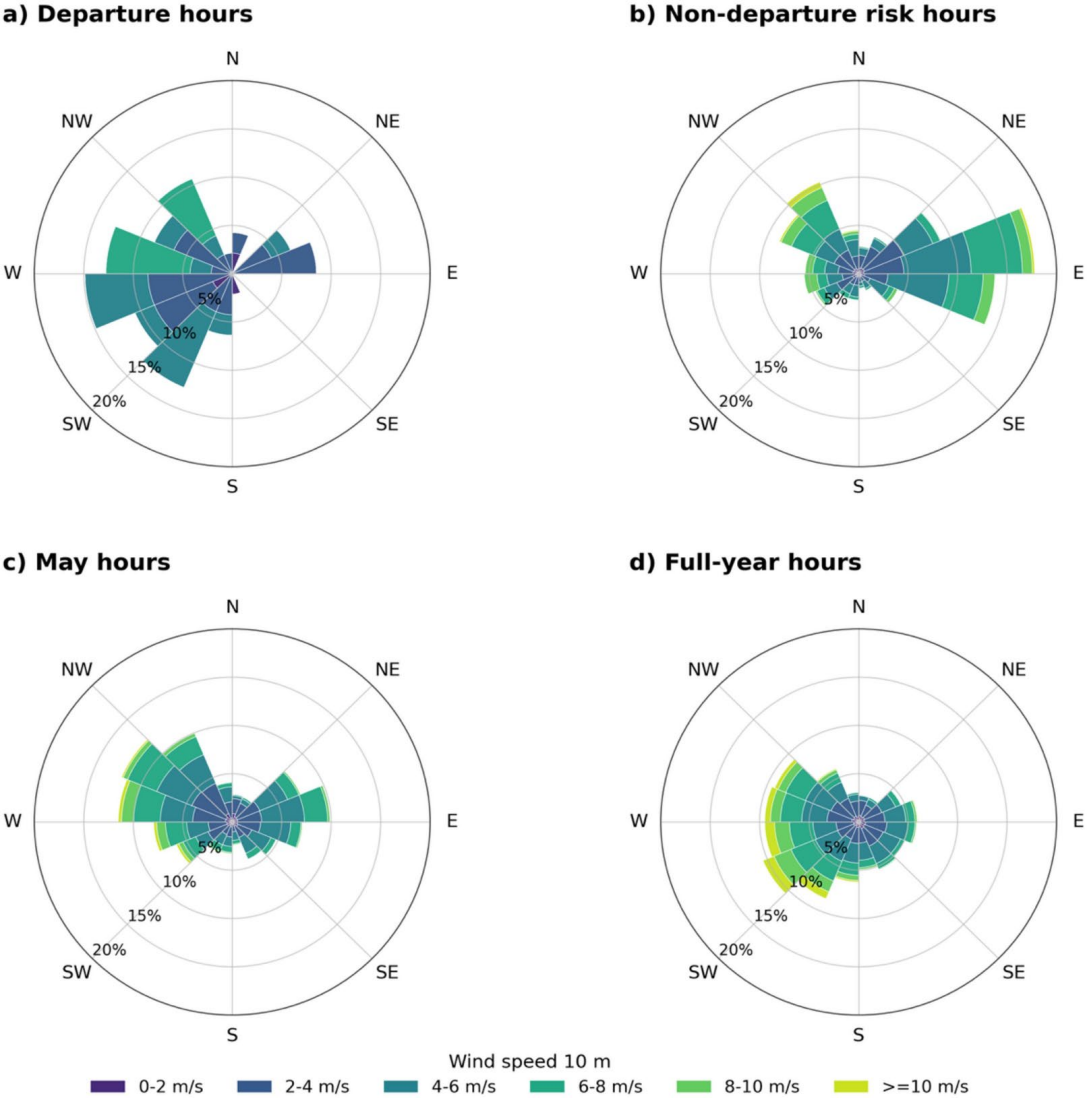
Wind conditions at individual Wadden Sea staging locations shown as wind roses, comparing departure hours against background wind distributions to confirm selective departure under favourable conditions. Panels show the relative frequency of wind direction and speed (m/s, colour‐coded) for: (a) departure hours (*n* = 47), (b) non‐departure hours within the spring departure window (14 May–6 June; *n* = 17,989), (c) all available hours within the departure window (*n* = 18,036), and (d) all hours across the full annual cycle. Departure hour is defined as the last complete hour before the GPS‐classified departure timestamp. Positive wind assistance values indicate tailwind support along the 47° mean migration bearing.

During the spring migration period, westerly and north‐westerly ground winds predominated, frequently providing favourable along‐wind conditions for north‐eastward migration, consistent with the prevailing jet‐stream orientation over the Wadden Sea region (Figure 5). At the hour of departure, mean wind assistance along the 47° mean departure bearing was +1.76 m/s at 100 m altitude, with 70% of departures occurring under positive wind assistance (tailwind conditions). This contrasts with the background distribution across all available hours in the staging window, where mean wind assistance was −0.64 m/s and only 43% of hours had positive assistance, confirming active selection for favourable wind conditions relative to background availability (Figure 5).

### 3.3 Weather Drivers of Departure Timing

Weather conditions during the main migration window (14 May–6 June) varied among years but consistently provided opportunities for supportive winds, with relative humidity ranging from 26% to 100% (IQR 65%–85%) and atmospheric pressure from 999 to 1039 hPa (IQR 1011–1020 hPa). Precipitation in the 3 h before departure was minimal across all 47 events (mean = 0.07 ± 0.06 mm SE; maximum = 2.8 mm; 89% of departure hours recorded zero precipitation) and was therefore excluded as a predictor, as the observed values were unlikely to constitute a meaningful constraint on take‐off decisions (Table 2). Cox proportional hazards analyses, based on 47 departure events, revealed a clear hierarchy of model support (Table 3). The best‐supported model combined wind assistance at 100 m and atmospheric pressure (ΔAIC_c_ = 0), outperforming the next‐best model combining wind assistance and relative humidity (ΔAIC_c_ = 3.5). All remaining models received substantially lower support (ΔAIC_c_ ≥ 5), indicating that departure decisions were primarily governed by the joint influence of directional wind support and synoptic pressure conditions.

**TABLE 2:** Summary of meteorological variables considered in proportional hazards modelling.

Variable	Unit	Window	Biological interpretation	Outcome
Wind assistance (100 m)	m/s	Instantaneous	Tailwind component along 47° bearing	In best model (wind_pres); primary predictor
Wind assistance (10 m)	m/s	Instantaneous	As above at near‐surface	Sensitivity analysis only
Atmospheric pressure	hPa	Instantaneous	Synoptic stability indicator	In best model (wind_pres)
Relative humidity	%	Instantaneous	Air moisture, proxy for visibility	In second‐ranked model (wind_rH)
Dewpoint temperature	°C	Instantaneous	Air moisture	Tested; low model support (ΔAIC_c_ ≥ 5)
Cloud cover	fraction	Instantaneous	Visibility and ceiling height	Tested; low model support (ΔAIC_c_ ≥ 5)
Dewpoint trend (12 h)	°C/h	12 h rolling OLS	Moisture dynamics	Tested; low model support (ΔAIC_c_ ≥ 5)
Pressure trend (24 h)	hPa/h	24 h rolling OLS	Direction of synoptic change	Tested; low model support (ΔAIC_c_ ≥ 5)
Wind assist trend (12 h)	m/s/h	12 h rolling OLS	Strengthening/weakening flow	Tested; low model support (ΔAIC_c_ ≥ 5)
Temperature (2 m)	°C	Instantaneous	Thermal conditions	Excluded before modelling—collinear (VIF > 100)
Precipitation (3 h sum)	mm	3 h prior	Pre‐flight rain exposure	Excluded before modelling—negligible (89% zero)

*Note:* Hourly weather was extracted at each individual's last GPS‐defined staging location prior to departure. Trend variables were derived as rolling ordinary least‐squares regression slopes over the preceding 12 h (pressure: 24 h).

**TABLE 3:** Model ranking of Cox proportional hazards candidates (100 m wind assistance as primary predictor).

Model	Included_predictors	*k*	LogLik	ΔAIC_c_	PH global *p*
wind_pres	Wind assistance + atmospheric pressure	2	−126	0	0.039
wind_rH	Wind assistance + relative humidity	2	−127.7	3.5	0.009
tailwind	Tailwind binary	1	−129.6	5	0.033
wind_rH_pt24	Wind assistance + relative humidity + pressure trend (24 h)	3	−127.3	5	0.008
wind_rH_dewpt	Wind assistance + relative humidity + dewpoint	3	−127.4	5.1	0.023
wind_rH_cloud	Wind assistance + relative humidity + cloud cover	3	−127.4	5.2	0.008
wind_dt12	Wind assistance + dewpoint trend (12 h)	2	−128.7	5.5	0.115
wind_dewpt	Wind assistance + dewpoint	2	−128.8	5.6	0.079
wind	Wind assistance only	1	−130.5	6.8	0.014
wind_cloud	Wind assistance + cloud cover	2	−129.8	7.7	0.017
wind_pt24	Wind assistance + pressure trend (24 h)	2	−130.1	8.3	0.014
wind_wt12	Wind assistance + wind‐assistance trend (12 h)	2	−130.4	8.9	0.002
wind_trend	Wind‐assistance trend only	1	−135.6	17	0.123
pressure	Atmospheric pressure only	1	−135.6	17.2	0.289
null	Intercept only	0	−136.8	17.4	_
dewpt_trend	Dewpoint trend only	1	−135.8	17.6	0.909
pres_trend	Pressure trend only	1	−135.9	17.7	0.006
dewpt	Dewpoint only	1	−136	17.8	0.383
humidity	Relative humidity only	1	−136.4	18.6	0.017
cloud	Cloud cover only	1	−136.7	19.4	0.035

*Note:* *k* = number of estimated parameters; LogLik = log‐likelihood of the fitted model; ΔAIC_c_ = difference in Akaike's information criterion corrected for small sample size (*n* = 47 departure events) relative to the best‐supported model, ΔAIC_c_ < 2 are considered equivalent; PH global *p* = *p*‐value from the global Schoenfeld residual test for proportional hazards, testing whether any predictor in the model violates the assumption of a constant hazard ratio over time.

**FIGURE 6:**
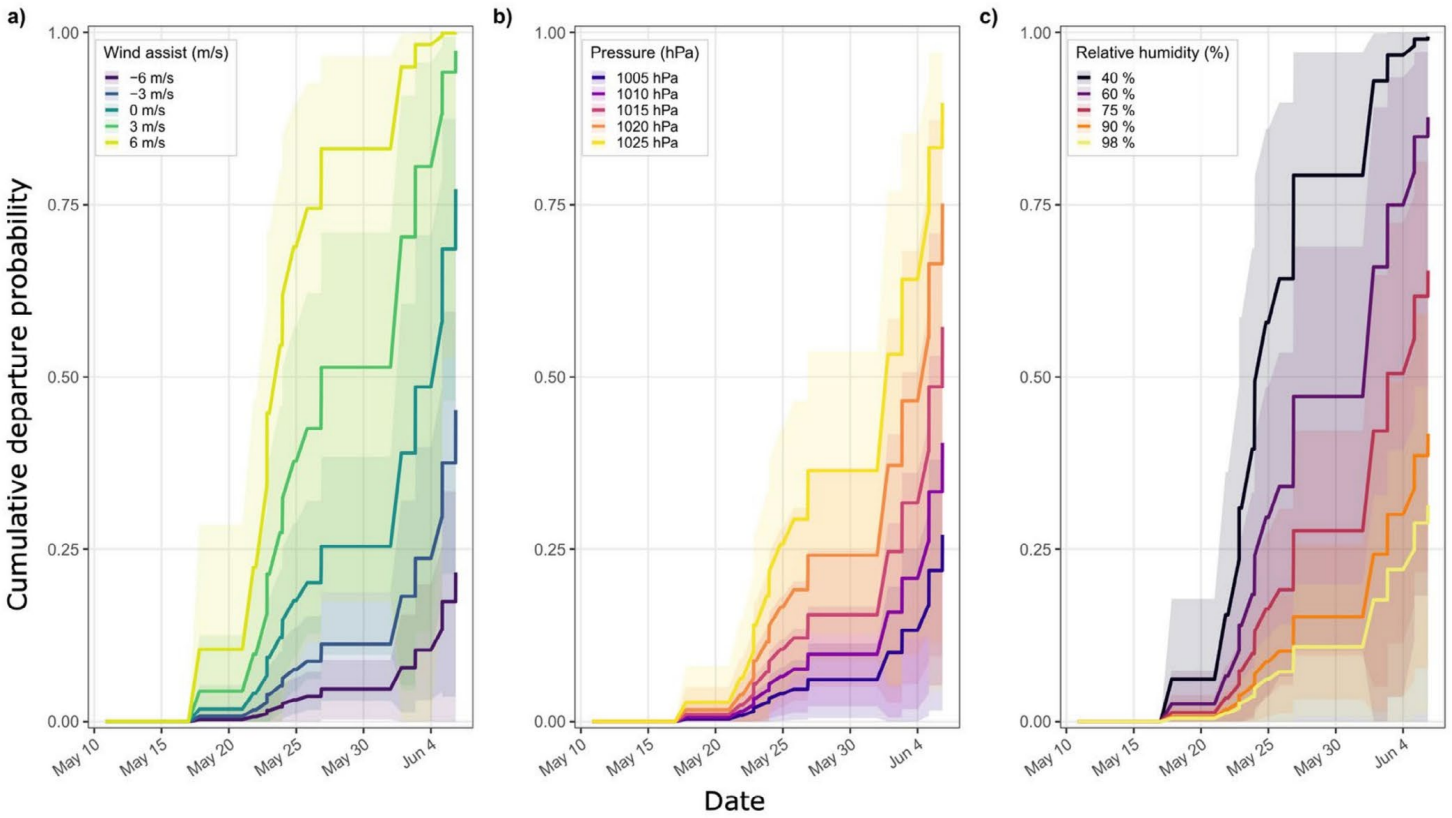
Predicted departure probability (1 − *S*(*t*)) derived from Cox proportional hazards models for Brent Geese. (a) Wind assistance along the 47° migration bearing from −6 to +6 m/s at median atmospheric pressure; (b) atmospheric pressure from 1005 to 1025 hPa at median wind assistance; (c) relative humidity from 40% to 98% at median wind assistance. Shaded areas denote 95% confidence intervals. Positive values indicate tailwind assistance along the migration bearing.

Within the best‐supported model, both predictors exerted strong effects. Departure probability increased steeply with increasing wind assistance (HR = 1.20, 95% CI 1.12–1.29) and with increasing atmospheric pressure (HR = 1.10, 95% CI 1.06–1.15). In the second‐ranked model, relative humidity independently reduced departure probability (HR = 0.959, 95% CI 0.932–0.986) net of wind conditions, with wind assistance showing a consistent effect (HR = 1.18, 95% CI 1.10–1.26). These relationships are illustrated in Figure 6, where cumulative departure probability accelerated rapidly under strong tailwinds and high pressure but remained nearly constant under calm or low‐pressure conditions. Wind assistance represents a local predictor extracted at each bird's GPS‐defined staging location, while atmospheric pressure represents a large‐scale synoptic variable, indicating that departure decisions integrate meteorological information across spatial scales simultaneously.

### 3.4 Proportional Hazards Evaluation and Temporal Dynamics

**FIGURE 7:**
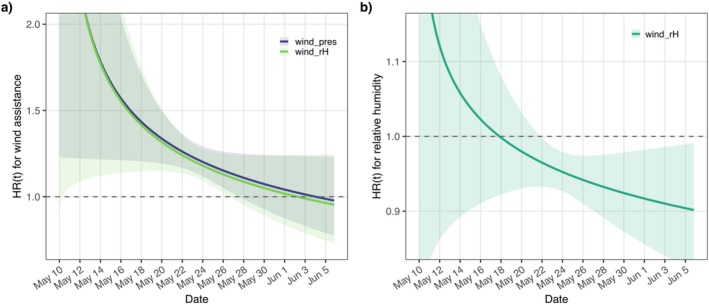
Time‐dependent hazard ratio HR(*t*) derived from time‐varying‐coefficient Cox models. (a) HR(*t*) for wind assistance per +1 m/s tailwind, shown for both top‐ranked models (wind + pressure and wind + humidity) to demonstrate robustness of the temporal pattern across model specifications. (b) HR(*t*) for relative humidity per +1% from the wind + humidity model. Dashed horizontal line indicates HR(*t*) = 1 (no effect on departure probability). Shaded areas indicate 95% confidence intervals. Atmospheric pressure showed no time‐varying effect (Table 4) and is therefore not shown as an HR(*t*) curve.

Formal tests of the proportional hazards assumption indicated that the influence of wind assistance varied over time (Table 4). In the best‐supported wind + pressure model, the global Schoenfeld test showed a significant deviation from proportionality (*p* = 0.039), and including a TVC for wind assistance improved model fit (likelihood‐ratio test: $\chi_{1}^{2}$ = 3.95, *p* = 0.047; ΔAIC_c_ = −1.67). A stronger improvement was observed in the wind + humidity model ($\chi_{1}^{2}$ = 7.22, *p* = 0.007; ΔAIC_c_ = −4.93). In contrast, atmospheric pressure showed no time‐dependent effect ($\chi_{1}^{2}$ = 0.49, *p* = 0.486; ΔAIC_c_ = +1.80). Relative humidity also showed time‐dependence ($\chi_{1}^{2}$ = 4.73, *p* = 0.030; ΔAIC_c_ = −2.44). The estimated HR(*t*) for wind assistance (Figure 7a) revealed a pronounced early‐season advantage of supportive winds that gradually weakened towards the end of the risk period: at approximately 14 May (hour 115) the hazard was increased approximately 1.7‐fold per +1 m/s tailwind (HR = 1.69, 95% CI 1.22–2.34), moderating to HR = 1.16 (95% CI 1.06–1.26) by approximately 26 May (hour 380), and approaching unity by early June (HR = 0.978, 95% CI 0.776–1.23, hour 644). Results from the wind + humidity model were closely consistent at the same timepoints, confirming the robustness of this temporal pattern across model specifications (Figure 7b).

**TABLE 4:** Proportional hazards and time‐varying‐coefficient (TVC) diagnostics for key models.

Model	Variable tested	PH global *p*	LRT *χ* ^2^	LRT *p*	ΔAIC_c_	PH interpretation
		(Schoenfeld)	(df = 1)		(TVC − static)	
wind_pres	Wind assistance	0.039	3.95	0.047	−1.67	PH violated; time‐dependent effect retained
wind_pres	Atmospheric pressure	0.039	0.49	0.486	+1.80	PH satisfied; static effect retained
wind_rH	Wind assistance	0.009	7.22	0.007	−4.93	PH violated; time‐dependent effect retained
wind_rH	Relative humidity	0.009	4.73	0.030	−2.44	PH violated; time‐dependent effect retained

*Note:* AIC_c_ = Akaike information criterion corrected for small sample size. PH global *p* = global Schoenfeld residual test (cox.zph). LRT = likelihood‐ratio test (df = 1) comparing the static model to a time‐varying‐coefficient (TVC) model, *tt*(*x*) = *x* × log(1 + *t*). ΔAIC_c_ < 0 with LRT *p* < 0.05 indicates the TVC model improves fit, that is, the effect changes over the season.

The non‐parametric baseline hazard, estimated from a stratified null model, increased monotonically across all four time‐of‐day strata, rising from near zero in early May to mean cumulative hazard values of 0.18–0.78 by early June, representing the statistical signature of increasing departure tendency independent of weather conditions. As a descriptive check on the TVC result, mean tailwind at departure declined from 3.2 m/s early in the window (before approximately 23 May) to 1.6 m/s mid‐season and 0.5 m/s late, consistent with the model‐based evidence for declining wind selectivity, though the Kruskal–Wallis test across timing groups was not significant (*χ* ^2^ = 2.73, df = 2, *p* = 0.256) due to low power with approximately 16 birds per group. Pressure showed no significant variation across timing groups (Kruskal–Wallis *p* = 0.684), consistent with its role as a stable background quality filter rather than an urgency‐modulated cue. Humidity likewise showed no significant variation in this descriptive check (Kruskal–Wallis *p* = 0.372), although the model‐based TVC analysis indicated that its effect on departure probability did change over the season (see above); the discrepancy likely reflects the lower power of the group‐wise comparison relative to the full Cox model.

## 4 Discussion

This study provides new insights into the proximate environmental triggers of spring migration in Brent Geese, combining high‐resolution individual‐level GPS tracking data with hourly meteorological data extracted at each bird's staging location and time‐to‐event modelling. Wind assistance emerged as the dominant cue governing departure timing, with atmospheric pressure acting as an independent secondary predictor and relative humidity competitive in the second‐ranked model. Spring migration represents a critical phase in the annual cycle of geese and other Anatidae, through direct influences on subsequent reproductive performance (Arzel et al. 2006), yet quantitative studies on the proximate meteorological drivers of spring departure in this group have remained scarce.

Three questions that have remained unresolved for Brent Geese departing the Wadden Sea are addressed by these findings. First, while prior work identified wind as a general correlate of departure in this population (Green et al. 2002), the relative importance of multiple simultaneous meteorological variables at the individual level had not been formally quantified; individual‐level point weather extraction removes the exposure misclassification inherent in regional grid analyses and reveals pressure and humidity as distinct secondary cues alongside wind. Second, whether wind selectivity declines over the departure window—as predicted by time‐minimisation theory (Åkesson and Hedenström 2000)—had not been tested explicitly for this species; the time‐varying coefficient analysis confirms this decline, with wind selectivity strongest in mid‐May and statistically indistinguishable from zero by early June. Third, no previous analysis of departure timing in this system had applied a framework separating the statistical signature of seasonal time pressure from weather‐driven variation; the Cox proportional hazards approach achieves this separation through the non‐parametric baseline hazard, a distinction unavailable to simpler regression approaches and essential for correctly attributing effects to meteorological rather than phenological drivers. Together, these variables explained much of the observed variability in departure behaviour, indicating an adaptive migratory strategy enabling geese to exploit short‐term atmospheric opportunities within the narrow spring window.

Brent Geese timed their departures to coincide with supportive winds, avoiding headwinds and departing when along‐wind flow aligned with their north‐eastward migratory direction. Within the seasonally constrained departure window, largely set by endogenous timing and photoperiod, geese appeared to use short‐term wind and pressure conditions at their individual staging locations to determine the exact timing of departure. This behaviour mirrors earlier evidence across species showing that favourable winds reduced energetic costs, shortened flight duration and enhanced migration efficiency (Liechti 2006; Shamoun‐Baranes et al. 2010). In Brent Geese, satellite‐tracked individuals consistently selected migration days with tailwinds along their route (Green et al. 2002), and Greenland White‐fronted Geese ( *Anser albifrons flavirostris* , Audubon 1839) similarly delayed their departure until supportive winds prevailed (Fox et al. 2003). This strategy aligns with studies showing that flight performance in Brent Geese operated close to their physiological limits, requiring strategic use of tailwinds and stepwise altitude gains during major crossings (Pennycuick 1996). Comparable physiological constraints have been shown to link weather and energetic limitations across migration stages (Newton 2004, 2006), suggesting that atmospheric opportunities directly influence both individual performance and population dynamics. Earlier reviews highlighted the lack of quantitative studies on spring‐staging Anatidae (Arzel et al. 2006), and the present study helps to fill this gap by explicitly modelling the proximate meteorological drivers of departure decisions. Comparable responses to synoptic‐scale wind patterns have been reported across geese and other waterfowl, suggesting a general strategy in which migrants balance time, energy and safety to maximise fitness returns (Kölzsch et al. 2016). Even for large, experienced migrants, departure decisions remain finely tuned to external atmospheric conditions rather than being governed solely by endogenous schedules, particularly in flapping species where the energetic costs increase in line with body mass (Elliott 2016).

The independent positive effect of atmospheric pressure on departure probability—stable across the season and requiring no time‐varying coefficient—indicates that birds respond to synoptic‐scale conditions beyond instantaneous wind. High atmospheric pressure is associated with anticyclonic circulation, clear skies, reduced precipitation risk, and more predictable wind fields, all of which reduce the energetic and navigational costs of initiating a long‐distance crossing. Pressure‐dependent departure has been documented in a range of migratory species, from passerines to raptors (Cooper et al. 2023; Shamoun‐Baranes et al. 2006), but its independent quantification alongside wind in a waterfowl species using individual‐level data is novel. This finding adds to a growing body of evidence that migrants integrate multiple simultaneous meteorological signals rather than responding to a single dominant cue (Cooper et al. 2023). The distinction between wind assistance—a local, rapidly fluctuating variable—and atmospheric pressure—a large‐scale, slowly varying synoptic indicator—suggests that departure decisions integrate meteorological information across spatial and temporal scales simultaneously: pressure providing a background signal of overall suitability, and wind providing the immediate short‐term trigger for take‐off. The absence of any time‐varying pressure effect further distinguishes it from wind: whereas wind selectivity declined over the season as time pressure mounted, the pressure response remained constant throughout, consistent with pressure acting as a stable quality filter rather than an urgency‐modulated cue.

The inverse association with relative humidity likely reflects avoidance of fog, drizzle, or low‐visibility conditions that can impair flock coordination and orientation, while dry, stable air masses generally coincide with clearer skies and calmer atmospheric layers, providing both visual and aerodynamic advantages (Alerstam and Lindström 1990; Panuccio et al. 2019). This interpretation is supported by radar and telemetry studies showing that migrants often delayed departure under humid or overcast conditions (Sjöberg et al. 2015). Humidity thus appears to act as a real but secondary cue, reflecting the overall quality of the atmospheric environment rather than imposing a direct energetic constraint on flight, and its influence was less consistent over the season than the time‐varying wind effect.

The relationship between departure probability and wind assistance observed here aligns with general patterns across migratory taxa. Using similar event‐based models, Dossman et al. (2016) demonstrated that both tailwind assistance and fuel stores strongly predicted departure likelihood in passerine migrants, while Cooper et al. (2023) confirmed that Cox proportional hazards models effectively quantified how instantaneous departure probability varied with changing weather conditions. Together, these findings indicate that time‐to‐event frameworks capture a fundamental behavioural rule of migration, with departures timed to maximise efficiency under transient atmospheric opportunities.

The time‐varying coefficient analysis revealed that the effect of wind assistance declined as the season progressed. Early in the season, when sufficient time remained to reach the Arctic breeding grounds, individuals appeared to wait for optimal conditions and departed almost exclusively during strong tailwinds. Towards the end of the window, however, departures became less selective, suggesting that increasing migratory urgency and physiological readiness progressively outweighed environmental constraints (Clausen et al. 2003; Ebbinge and Spaans 1995). This temporal decline was consistent across both top‐ranked models, confirming its robustness. Such urgency reflects the demographic trade‐offs typical of species where adult timing decisions are shaped by offspring requirements in distant breeding habitats (Fokkema et al. 2020). The weakening of the wind effect over time therefore represents a biologically meaningful pattern, rather than a statistical artefact, illustrating flexible, context‐dependent departure thresholds that directly affect reproductive success (Clausen et al. 2003). Sex did not emerge as a significant predictor of departure timing, consistent with the family‐group migration structure typical of geese, in which paired individuals depart together and sex‐specific scheduling is therefore unlikely (Kölzsch et al. 2020).

The increasing baseline hazard over the season represents the statistical signature of time pressure building independently of weather conditions, and all‐weather effects reported here are estimated conditional on this background trend. Descriptively, the decline in mean wind conditions at departure from early to late season is consistent with the model‐based evidence, though statistical power at the level of individual timing comparisons was limited. Individual staging duration in the Wadden Sea could not be formally included as a covariate because complete arrival dates were unavailable for a substantial proportion of birds; however, the non‐parametric baseline hazard absorbs any systematic increase in departure probability over the season regardless of its cause. Together, these results support a hierarchical decision framework in which internal migratory readiness defines the seasonal window for departure, while short‐term atmospheric cues modulate the exact timing within that window (Cooper et al. 2023).

Departures occurred mainly in the late afternoon and evening, suggesting that the geese synchronised their take‐off with periods of favourable wind and thermal stability while maintaining sufficient daylight for visual coordination and flock formation, as also observed in Eurasian Curlews ( *Numenius arquata* , Linnaeus 1758) (Schwemmer et al. 2021). Unlike smaller nocturnal migrants that rely on night‐time conditions for navigation and predator avoidance, Brent Geese are largely diurnal and highly social (Berdahl et al. 2018; Kharitonov et al. 2016). Daylight departures likely facilitate visual coordination and spatial alignment within large flocks (Åkesson and Hedenström 2007) and may allow exploitation of a less turbulent atmosphere during evening calm (Alerstam 2009). The wide variation in distances covered within the first 24 h after departure further suggests that individuals modulate their flight effort according to internal and external cues (Green et al. 2002). Because the first migration leg requires crossing the Baltic Sea, which presents a broad ecological challenge with limited opportunities for rest or refuelling, it is critical to select appropriate departure conditions for a successful and energetically efficient passage. Comparable long‐distance barrier crossings, such as over the Greenland icecap, have been shown to push Brent Geese to their biomechanical limits, highlighting the importance of strong wind support (Gudmundsson et al. 1995).

The strong linkage between wind conditions and migration timing also has direct implications for conservation management. The main migration corridor from the Wadden Sea towards the Russian Arctic overlaps with zones of intensive offshore wind‐energy development in the North and Baltic Seas (4C Offshore 2026). The concentration of departures into short, meteorologically defined windows implies that migration intensity is temporally clustered. Temporary turbine curtailment during these forecastable peak periods—identifiable in advance by integrating departure models with real‐time weather forecasts—could substantially reduce collision risk while minimising the overall duration of operational restrictions (Schwemmer et al. 2023). Similar forecasting approaches could inform flight safety management along the flyway even more precisely, as large‐scale goose departures within narrow meteorological windows may increase collision risk with aircraft in busy airspace corridors, where migration‐based warning systems have been shown to reduce damaging bird strikes by up to 45% (van Gasteren et al. 2019). Integrating departure models with real‐time weather forecasts would allow managers to anticipate high‐risk migration windows and minimise collision risks, while limiting the duration of restraint measures. More broadly, the dependence on wind cues highlights the potential sensitivity of this migration system to climatic shifts. Changes in North Atlantic wind regimes and jet‐stream positioning may alter the timing and availability of supportive winds, with downstream effects on migration schedules and breeding phenology (Bathrick et al. 2024; Van Eerden et al. 2005). Continued monitoring of weather conditions and departure behaviours will be essential to anticipate potential mismatches between environmental cues and migratory readiness. Long‐term tracking of migration phenology and body condition, as advocated by Piersma and Lindström (2004), may thus provide an early‐warning system for environmental change within Arctic–temperate flyways.

Applying Cox proportional hazards models to high‐resolution tracking data offers a robust framework for analysing time‐dependent behavioural decisions in migratory birds. The counting‐process structure and inclusion of time‐varying predictors provide a detailed understanding of how external drivers interact with internal state to shape movement decisions, and the non‐parametric baseline hazard separates background seasonal urgency from weather‐driven variation—a distinction that simpler regression approaches cannot achieve. Such event‐based models quantify how the instantaneous probability of departure varies with environmental and internal cues, making them particularly suitable for studying dynamic weather responses in migration ecology (Cooper et al. 2023). Future work could extend this approach by incorporating additional external cues, such as green‐wave progression or snowmelt dynamics (Curk et al. 2020), as well as individual‐level physiological measurements. Such analyses could explicitly test the green‐wave hypothesis (van der Graaf et al. 2006), which predicts that herbivorous waterfowl track successive peaks in forage quality and nutrient availability along climatic gradients from the Wadden Sea to Arctic breeding grounds. Recent advances in harness attachment methods and logger technology are likely to enable tracking of more individuals across multiple years, facilitating tests of behavioural consistency and individual divergence in weather‐dependent departure strategies.

These findings reveal that Brent Geese employ a flexible but strongly weather‐dependent strategy for initiating spring migration, one that becomes progressively less selective as the breeding deadline approaches. This temporal dynamic illustrates the hierarchical nature of migratory decision‐making: endogenous rhythms define the seasonal window, large‐scale synoptic conditions define its overall quality, and local atmospheric conditions provide the immediate trigger for take‐off. The pronounced responsiveness of departure decisions to transient meteorological conditions highlights both the behavioural sophistication of these migrants and their potential vulnerability to projected changes in North Atlantic wind regimes and pressure patterns driven by ongoing climate change.

## Author Contributions

**Nora Theurich:** conceptualization (equal), data curation (lead), formal analysis (lead), investigation (lead), methodology (lead), software (lead), validation (equal), visualization (lead), writing – original draft (lead), writing – review and editing (equal). **Frédéric Jiguet:** validation (equal), writing – review and editing (equal). **Pierrick Bocher:** validation (equal), writing – review and editing (equal). **Stefan Garthe:** data curation (supporting), funding acquisition (equal), project administration (equal), validation (equal), writing – review and editing (equal). **Philipp Schwemmer:** funding acquisition (equal), project administration (equal), supervision (lead), validation (equal), writing – review and editing (equal).

## Funding

Financial support was received from the German Federal Agency for Nature Conservation (BfN) with funds from the Federal Ministry for the Environment, Climate Action, Nature Conservation and Nuclear Safety (BMUKN) through the projects BIRDMOVE (FKZ 3515822100), TRACKBIRD (FKZ 3519861400), OWP‐Vogelzug (FKZ 352315100A) and within the project Tricma^2^ (FKZ 03 F0960 C) funded by the Federal Ministry of Research, Technology and Space (BMFTR).

## Conflicts of Interest

The authors declare no conflicts of interest.

## Data Availability

Data on the geese tagged in Germany are available at movebank.org under the study reference ID 2065254122. Animal welfare licences were granted by the Ministerium für Energiewende, Klimaschutz, Umwelt und Natur of the federal state of Schleswig‐Holstein (file number: V 242‐39334/2022 (41‐5/22), V 241‐35852/2017 (88‐7/17), and V 312‐7224.121‐37 (42‐3/13)). Data on the geese tagged in France are available on the movebank.org platform under the study reference ID 2529657945, while capture, ringing, and tagging were authorised by the national authority MNHN‐CRBPO under the reference file PP1275.
